# PinX1 Is a Potential Prognostic Factor for Non-Small-Cell Lung Cancer and Inhibits Cell Proliferation and Migration

**DOI:** 10.1155/2017/7956437

**Published:** 2017-07-26

**Authors:** Shengguang Wang, Hua Zhang, Jianquan Zhu, Chenguang Li, Jinfang Zhu, Bowen Shi, Bin Zhang, Changli Wang

**Affiliations:** ^1^Department of Lung Cancer, Tianjin Medical University Cancer Institute and Hospital, Tianjin 300060, China; ^2^Tianjin Lung Cancer Center, Tianjin 300060, China; ^3^Tianjin Key Laboratory of Cancer Prevention and Therapy, Tianjin 300060, China; ^4^National Clinical Research Center for Cancer, Tianjin 300060, China

## Abstract

PinX1 has been identified as a suppressor of telomerase enzymatic activity. However, the tumour-suppressive roles of PinX1 in different types of human cancers are unclear. PinX1 expression status and its correlation with clinicopathological features in non-small-cell lung cancer (NSCLC) have not been investigated. Accordingly, in this study, we aimed to evaluate the roles of PinX1 in NSCLC. PinX1 expression status was examined by immunohistochemistry using tissue microarray from a total of 158 patients. Correlations among PinX1 expression, clinicopathological variables, and patient survival were analysed. Furthermore, we overexpressed PinX1 in NSCLC cells and tested telomerase activity using real-time quantitative telomeric repeat amplification protocol (qTRAP) assays. Proliferation and migration of NSCLC cells were examined using the MTS method, wound healing assays, and transwell assays, respectively. Our results showed that negative PinX1 expression was associated with a poor prognosis in NSCLC. Sex, smoking status, lymph gland status, subcarinal lymph node status, pathological stage, and PinX1 expression were related to survival. PinX1 was not an independent prognostic factor in NSCLC. PinX1 overexpression inhibited proliferation and migration in NSCLC cells by suppressing telomerase activity. Our findings suggested that PinX1 could be a potential tumour suppressor in NSCLC and that loss of PinX1 promoted NSCLC progression.

## 1. Introduction

Lung cancer (LC) is one of the most common types of malignant tumours, and the incidence of LC has increased in recent years. According to the latest data, the number of new cases of carcinoma of the lung and bronchus in the United States of America (USA) was expected to reach up to 224,390 in 2015, among which 158,080 patients were estimated to have died from their disease [1]. Additionally, the incidence and mortality rates of LC increased in 2015 [2]. In China, an estimated 4,292,000 new cancer cases and 2,814,000 cancer-related deaths occurred in China in 2015, with LC being the most common cancer type and the leading cause of cancer-related death [3]. LC is related to a variety of factors [4–8], and because of the major public health concerns associated with LC, particularly non-small-cell lung cancer (NSCLC, accounting for approximately 80% of total LC), studies of the aetiology and pathogenesis of LC are urgently needed. Recent studies have shown that genetic factors could be the major cause of NSCLC [9]. Additionally, accumulating evidence has suggested that some genetic factors can also enhance the effects of environmental carcinogens in susceptible populations.

As has been shown previously, tumorigenesis most often occurs owing to the presence of genetic mutations [10]. Mutations in key genes that control cell proliferation, cell cycle progression, differentiation, apoptosis, and other important cell functions are likely to cause tumorigenesis. Telomeres are regions of repetitive nucleotide sequences combined with proteins at each end of a chromosome; these units function to protect the end of the chromosome from deterioration or fusion with neighbouring chromosomes and have been shown to control the cell division cycle [11]. Certain lengths of telomeres are prerequisites of cell division [12, 13]. In some actively dividing cells, such as cancer cells, telomerase is activated, adds repetitive sequences at the end of telomeres, and promotes the continuation of cell division [14]. PIN2/TERF1-interacting telomerase inhibitor 1 (PinX1) is a nucleolar protein evolutionarily conserved from yeasts to humans and is known to function as a suppressor of telomerase enzymatic activity through C-terminal domain binding with telomerase reverse transcriptase (TERT) [15]. PinX1 downregulation results in poor prognosis in some cancers, including gastric cancer [16], prostate cancer [17, 18], ovarian cancer [19, 20], and breast cancer [21, 22]. Another study showed that PinX1 not only functions as a telomerase inhibitor but also stabilises telomerase and further protects telomeres. The same study also showed that PinX1 contributes to tumorigenicity in cancer cells [23], in contrast with other studies.

PinX1 expression status has been shown to be altered in many cancers. However, no studies have evaluated the expression and prognostic value of PinX1 in NSCLC. Therefore, in this study, we investigated the expression of PinX1 and the association of PinX1 expression with clinicopathological features and outcomes in NSCLC using tissue samples from 158 patients. Our findings provide important insights into the role of PinX1 in NSCLC progression.

## 2. Materials and Methods

### 2.1. Patients and Samples

The study was approved by the Ethics Committee of Tianjin Medical University Cancer Hospital. A total of 158 patients, including 57 patients with adenocarcinoma and 101 patients with squamous cell carcinoma (SCC), with a median age of 61 years (range: 40–77 years), were enrolled in the study group. All samples were from patients who underwent surgery for complete removal of cancer and were collected from February 2010 to June 2012. All patients provided written informed consent for participation in the study.

### 2.2. Immunohistochemical Staining

The evaluation of PinX1 staining was performed in a blinded, independent manner by two pathologists. Immunohistochemistry (IHC) was performed by standard operating procedures, and a semiquantitative scoring method according to intensity (no, very weak, intermediate, and strong staining); no staining and very weak staining were considered “negative,” whereas intermediate and strong staining were considered “positive.” A primary polyclonal anti-PinX1 antibody (1 : 200; Sigma, St. Louis, MO, USA) and polymer peroxidase-labelled secondary antibody (ZSGB-Bio, Beijing, China) were used in IHC, followed by staining using a DAB Horseradish Peroxidase Color Development Kit (ZSGB-Bio, Beijing, China) [24].

### 2.3. Cell Culture

Human lung adenocarcinoma A549 cells and human squamous cell lung carcinoma H520 cells, two commonly used cell lines in LC research, were cultured in 1640 medium containing foetal calf serum (Gibco/Life Technologies, Carlsbad, CA, USA) and 1% penicillin/streptomycin (Invitrogen, Carlsbad, CA, USA), as previously reported. Cells were passaged when they reached 80–90% confluence using 0.25% trypsin-ethylenediaminetetraacetic acid (EDTA; Gibco).

### 2.4. Lentivirus-Induced PinX1 Overexpression

PinX1 cDNA was subcloned into a lentiviral vector pSL6 as previously reported [23, 25]. The production of recombinant lentivirus and their use in cell infection were performed according to standard procedures [26–28]. Specifically, lentiviral particles were generated by transfection of 293T cells with plasmids encoding the vesicular stomatitis virus G envelope, gag-pol, and PinX1. Medium containing lentiviral particles was harvested 48 h after transfection, filtered (0.45 *μ*m), and frozen until use. NSCLC cell lines were transduced using viral supernatants. The expression of PinX1 was confirmed by western blotting.

### 2.5. Cell Proliferation Assay

A One Solution Cell Proliferation Assay kit (the MTS/phenazine methosulphate [PMS] method; Promega, Madison, WI, USA) was used to assess cell proliferation according to the manufacturer's instructions. After incubation for 4 h at 37°C, the absorbance was measured using an automatic microplate reader (Gene Company, Hong Kong, China) at an optical density of 490 nm (OD_490_).

### 2.6. Telomeric Repeat Amplification Protocol (TRAP) Assay

Cells were lysed in CHAPS lysis buffer (United Chemi-Con, Rolling Meadows, IL, USA). Telomerase activity was quantified using a quantitative polymerase chain reaction- (qPCR-) telomeric repeat amplification protocol [29]. Real-time PCR was performed on an ABI PRISM 7500 Fast Sequence Detection System (Applied Biosystems, Foster City, CA, USA).

### 2.7. Wound Healing Assay

Cells were seeded into 6-well tissue culture plates. When cells reached 90–95% confluence as a monolayer, viral infection was carried out. The monolayer was slowly scratched with a new 0.2 mL pipette tip across the centre of the well. After scratching, the wells were gently washed twice with medium to remove the detached cells. Cells were then cultured for 48 h, and photographs were acquired (Olympus, Japan).

### 2.8. Transwell Migration Assay

Cell migration assays were performed using modified two-chamber plates with a pore size of 8 *μ*m. Cells with serum-free medium were seeded into the upper chamber of 24-well transwell plates. Medium containing 10% foetal bovine serum was added to the bottom chamber. Cells that migrated to the bottom of the filter were fixed, stained with crystal violet, and counted in nine random fields [30].

### 2.9. Statistical Analysis

Statistical analysis was performed using SPSS 20.0 software (SPSS, Inc., Chicago, IL, USA). The relationship between PinX1 protein expression and clinicopathological data in patients with NSCLC was estimated using chi-squared tests. The Kaplan-Meier method and the log-rank test were used to calculate overall survival (OS) and disease-free survival (DFS), and multivariate analyses were based on the Cox proportional hazards regression model for independent prognostic value. Differences with *P* values of less than 0.05 were considered statistically significant. Two-way analysis of variance was performed for evaluation of differences between groups, including control and PinX1-overexpression groups. The significance level was set to *α* = 0.05.

## 3. Results

### 3.1. Association between PinX1 Expression and Clinicopathological Features in Patients with NSCLC

**Figure 1:**
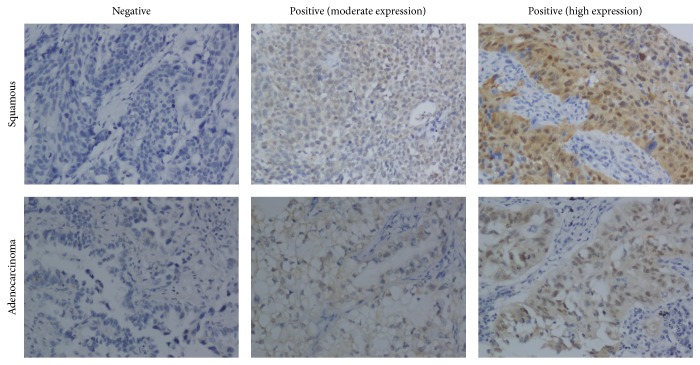
Expression of PinX1 in human NSCLC specimens. Magnification 200x.

PinX1 expression was examined using IHC in an NSCLC tissue microarray with 158 cancer samples (Figure 1). PinX1 positivity was detected in 41 of 158 (25.95%) NSCLC samples, while PinX1 negativity was observed in 117 of 158 (74.05%) samples. PinX1 expression was correlated with sex (*P* = 0.020, increased in men), smoking status (*P* = 0.034, increased in smokers), histological type (*P* < 0.001, increased in SCC), recurrence and metastasis after resection (*P* = 0.023, decreased in the presence of metastasis), and lymph node metastasis (*P* = 0.033, increased for N0). PinX1 expression was not associated with age, tumour location, operation method, T stage, or TNM stage (Table 1).

### 3.2. Association between PinX1 Protein Expression and Survival in Patients with NSCLC

**Figure 2:**
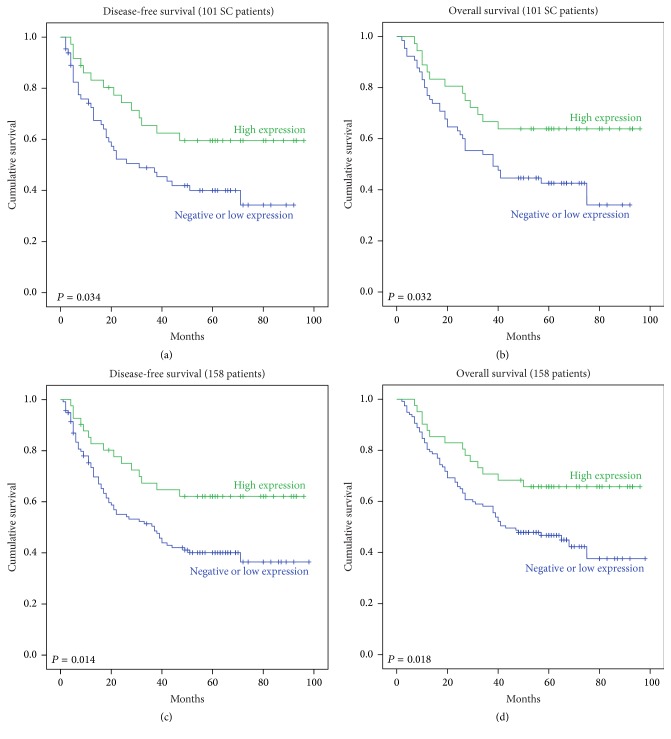
Negative PinX1 expression was correlated with poor overall survival and disease-free survival in NSCLC. (a) Correlations between PinX1 expression and 5-year disease-free cumulative survival in patients with the SCC subtype of NSCLC. (b) Correlations between PinX1 expression and 5-year overall cumulative survival in patients with the SCC subtype of NSCLC. (c) Correlations between PinX1 expression and 5-year disease-free cumulative survival in all patients with NSCLC. (d) Correlations between PinX1 expression and 5-year overall cumulative survival in all patients with NSCLC.

Kaplan-Meier survival curves were used to compare 5-year OS and DFS in all 158 patients and in 101 patients with the SCC subtype of NSCLC (Figure 2). The survival analysis showed that negative PinX1 was associated with poor OS (*P* = 0.032 for SCC and *P* = 0.018 for total) and DFS (*P* = 0.034 for SCC and *P* = 0.014 for total). For patients with SCC, the 5-year overall cumulative survival rate dropped from 64% in patients with positive PinX1 expression to 34% in those negative for PinX1 expression, and the 5-year disease-free cumulative survival rate dropped from 60% in patients with positive PinX1 expression to 34% in those negative for PinX1 expression (Figures 2(a) and 2(b)). In terms of 5-year OS in all patients, the cumulative survival function of positive PinX1 expression was 67%, while that of negative PinX1 expression was 37%; for 5-year DFS in all patients, the cumulative survival function of positive PinX1 expression was 62%, while that of negative PinX1 expression was 38% (Figures 2(c) and 2(d)).

Univariate Cox regression analysis showed that sex, smoking status, lymph gland status, subcarinal lymph node status, pathological stage, and PinX1 expression were related to survival (Table 2). Therefore, all of these variables were included in the multivariate analysis. Multivariate analysis revealed that PinX1 was not an independent prognostic factor in NSCLC (Table 3, *P* = 0.253 for DFS, *P* = 0.248 for OS).

### 3.3. PinX1 Overexpression Inhibited the Proliferation and Migration of NSCLC Cells

**Figure 3:**
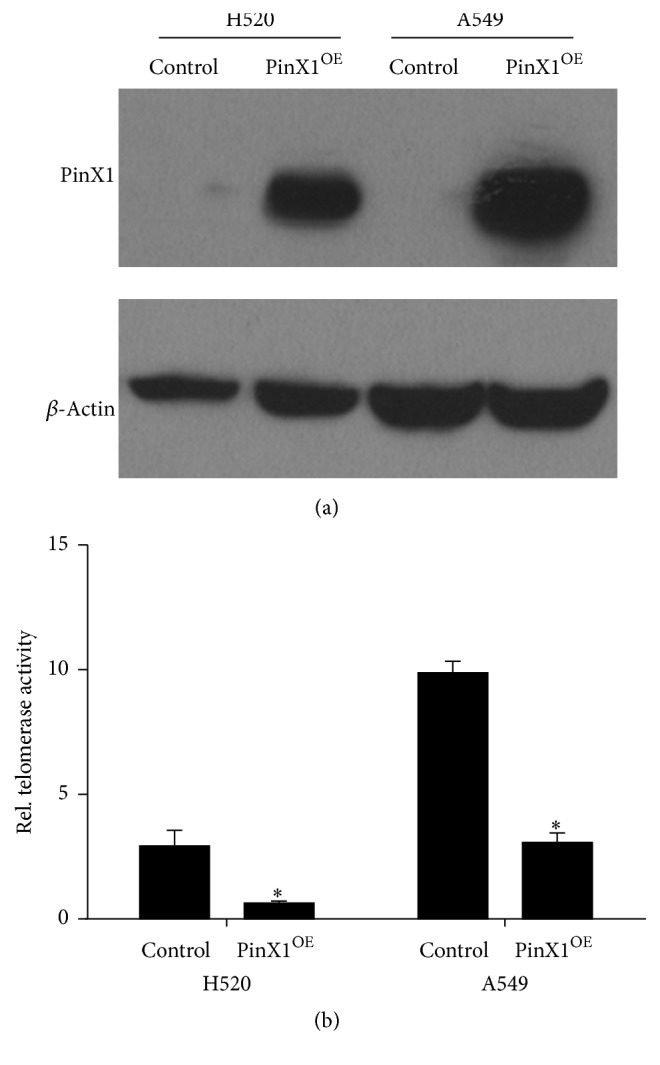
PinX1 overexpression decreased telomerase activity in both H520 and A549 cells. (a) PinX1 overexpression in both H520 and A549 cells. (b) Telomerase activity was evaluated in H520 and A549 cells with or without PinX1 overexpression. ^*∗*^*P* < 0.05. *n* = 9.

Because PinX1 was found to function as a tumour suppressor, we attempted to confirm our previous findings in NSCLC cells. We employed A549 and H520 cells derived from human NSCLC tissues and generated PinX1-overexpressing cells for both lines. PinX1 overexpression was confirmed by western blotting (Figure 3(a)). Since PinX1 is an inner telomerase inhibitor, we first detected the effects of PinX1 on telomerase activity in lung cancer cells. We tested telomerase activity and found that PinX1 overexpression inhibited telomerase activity in both A549 and H520 cells (Figure 3(b)).

**Figure 4:**
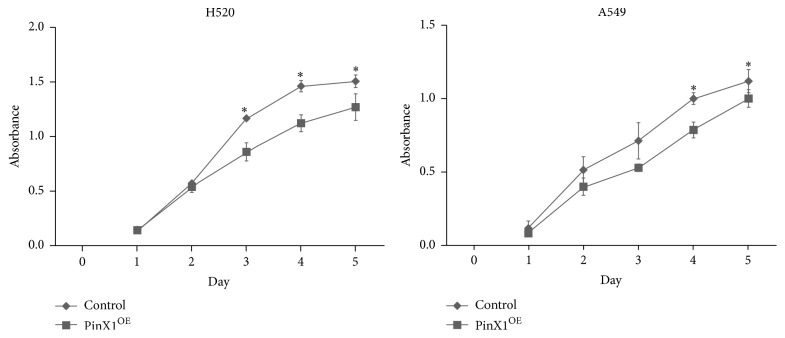
Cell proliferation was evaluated in H520 and A549 cells. ^*∗*^*P* < 0.05. *n* = 3 for H520 and A549 cells.

We then compared cell proliferation in PinX1-overexpressing cells and control cells for each cell line. H520 cells showed reduced proliferation when PinX1 was overexpressed beginning on day 3, whereas A549 cells showed reduced cell proliferation when PinX1 was overexpressed beginning on day 4 (Figure 4).

**Figure 5:**
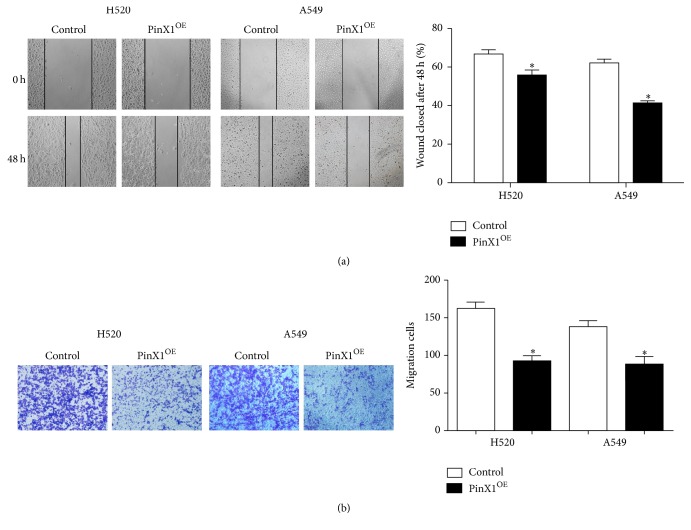
PinX1 overexpression inhibited cell migration in both H520 and A549 cells. (a) Wound healing assays in A549 (*n* = 8) and H520 (*n* = 9) cells with or without PinX1 overexpression after 48 h. ^*∗*^*P* < 0.05. (b) Transwell migration assay in LC cells with or without PinX1 overexpression. ^*∗*^*P* < 0.05. *n* = 9 for both lines.

Next, we evaluated cell migration with wound healing assays and transwell migration assays. In wound healing assays, both A549 and H520 cells showed reduced cell migration when PinX1 was overexpressed compared with that in control cells at 48 h (Figure 5(a)). We obtained similar results in transwell migration assays and found that both cell lines exhibited reduced signals with PinX1 overexpression, suggesting that PinX1 overexpression suppressed NSCLC cell migration (Figure 5(b)).

## 4. Discussion

### 4.1. Association between PinX1 Expression and Patient Survival in Cancers

Previous studies have suggested that PinX1 is an intrinsic telomerase inhibitor and a putative tumour-suppressor gene in human cancers. PinX1 expression is significantly reduced in a variety of cancer types. However, PinX1 expression has not been previously reported in NSCLC. In the present study, we enrolled a total of 158 patients with NSCLC and used IHC to detect PinX1 protein expression in tumour tissues. Among all cases, PinX1 positivity was found to be more frequent in SCC (36 of 101 samples) than in adenocarcinoma (5 of 55 samples), suggesting that PinX1 expression may be associated with specific tissue types.

Both the 5-year OS and 5-year DFS in patients with colorectal cancer are lower in patients with low/negative expression of PinX1 protein compared with that in patients with high/moderate expression of PinX1 [31] which is consistent with our current findings. In patients with SCC, PinX1 positivity was associated with a better prognosis, similar to the results in the total patient cohort. In other types of cancer, PinX1 expression has been shown to be a predictor of cervical SCC (CSCC) cell response to cisplatin/paclitaxel chemotherapy. Moreover, positive expression of PinX1 is correlated with the response of CSCC to cisplatin/paclitaxel chemotherapy and is an independent predictor of reduced survival [32]. In esophageal SCC (ESCC), reduced PinX1 expression did not affect ESCC cell response to 5-fluorouracil and cisplatin but did increase the efficacy of radiation therapy. High levels of PinX1 cause reduced cell death due to radiation and are a predictor of short disease-specific survival [32]. However, further studies are needed to assess the association between PinX1 expression and therapy effectiveness/sensitiveness, which could guide interventions and improve our understanding of NSCLC.

### 4.2. PinX1 Functioned as a Tumour-Suppressive Factor in NSCLC Cells

Our results revealed that PinX1 expression was related to survival but was not an independent prognostic factor, as evaluated by IHC using patient samples. Previous studies have shown that reduced expression of PinX1 is implicated in various human cancers, including breast cancer [21, 22], ovarian cancer [19, 20], gastric cancer [16], and liver cancer [31], suggesting that PinX1 may function as a tumour suppressor in multiple human cancers. In the present study, we also provided evidence of the tumour-suppressive role of PinX1 in NSCLC cells.

A549 cells were established by removal and culture of cancerous lung tissues in an explanted tumour from a 58-year-old Caucasian man. The cells produced were adenocarcinomatous alveolar basal epithelial cells. In contrast, H520 cells were established from an SCC of the lung and exhibited lower* p53* mRNA transcript expression than normal lung cells. In this study, tumour samples, including lung adenocarcinoma and squamous cell lung cancer, were analysed, and statistical analyses were performed with both histological types. As a result, we employed A549 (adenocarcinoma) and H520 (SCC) cells in subsequent experiments to support the statistical results. Our results showed that both cell lines lacked PinX1 expression, suggesting that these cell lines were good models for evaluating the effects of PinX1 in cells because of the low background PinX1 expression. In the current study, we overexpressed PinX1 in these two cell lines and attempted to determine the effects of PinX1 overexpression in these cells. Our results demonstrated that PinX1 overexpression resulted in reduced telomerase activity, consistent with previous studies [18, 19, 21].

We also confirmed that the proliferation and migration of the above-mentioned NSCLC cells were reduced by PinX1 overexpression compared with those in control cells, suggesting that PinX1 inhibited cancer development by suppressing telomerase activity. However, as a major limitation of our study, we were not able to identify any novel signalling pathways through which PinX1 could induce these effects. Nevertheless, our findings provide important contributions to our understanding of PinX1 function in cancer. Further studies are needed to fully elucidate the mechanisms through which PinX1 mediates NSCLC progression.

## Figures and Tables

**Table 1:** Correlations between PinX1 expression and clinicopathological parameters.

	PinX1		
	Positive	Negative	*P*
*Sex*			
Male	34	74	
Female	7	43	0.020
*Age*			
<60 years	19	52	
≥60 years	22	65	0.834
*Smoking status*			
Yes	34	76	
No	7	41	0.034
*Histology*			
Squamous	36	65	
Adenocarcinoma	5	52	0.000
*Lesion*			
Peripheral	28	83	
Central	13	34	0.750
*Tumour location*			
Left	17	48	
Right	24	69	0.961
*Resection type*			
Lobectomy	35	95	
Pneumonectomy	3	19	
Other	3	3	0.163
*Metastasis*			
Yes	15	67	
No	26	50	0.023
*T stage*			
T1	6	41	
T2 + T3	35	76	0.013
*N stage*			
N0	46	24	
N1 + N2	17	71	0.033
*TNM stage*			
I	13	31	
II	17	32	
III	11	54	0.082

**Table 2:** Univariate analysis with regard to DFS and OS.

	DFS			OS		
	*P* value	HR	95% CI	*P* value	HR	95% CI
Sex (male, female)	0.008	1.836	1.175–2.871	0.008	1.827	1.169–2.854
Age (<60 years, ≥61 years)	0.629	1.115	0.716–1.738	0.463	1.181	0.758–1.840
Smoking status (no, yes)	0.029	0.604	0.385–0.949	0.021	0.588	0.375–0.923
Histology (squamous, adenocarcinoma)	0.617	0.887	0.554–1.420	0.526	0.859	0.536–1.375
Surgical procedure (pneumonectomy, lobectomy, other)	0.196	1.297	0.874–1.924	0.109	1.379	0.931–2.403
Lesion (peripheral, central)	0.079	0.661	0.417–1.048	0.063	0.646	0.408–1.023
Tumour location (left, right)	0.466	0.848	0.545–1.320	0.431	0837	0.538–1.303
N0/N1-2	0.000	3.605	2.166–6.001	0.000	3.376	2.030–5.612
Subcarinal lymph node metastasis (positive, negative)	0.000	4.035	2.528–6.443	0.000	3.586	2.247–5.721
Pathological stage (I, II, IIIA)	0.000	1.710	1.417–2.065	0.000	1.673	1.387–2.018
PinX1 (positive, negative)	0.019	2.003	1.122–3.573	0.021	1.976	1.107–3.528

DFS, disease-free survival; OS, overall survival; HR, hazard ratio; CI, confidence interval.

**Table 3:** Multivariate analysis with regard to DFS and OS.

	DFS			OS		
	*P* value	HR	95% CI	*P* value	HR	95% CI
Sex (male, female)	0.430	1.279	0.694–2.356	0.451	1.281	0.673–2.440
Smoking status (yes, no)	0.580	0.838	0.448–1.568	0.449	0.777	0.404–1.494
Pathological stage (I, II, IIIA)	0.035	1.418	1.024–1.962	0.024	1.453	1.050–2.011
Pinx1 (negative, positive)	0.253	0.701	0.381–1.290	0.248	0.698	0.380–1.284
Subcarinal lymph node metastasis (positive, negative)	0.180	1.518	0.825–2.794	0.442	1.277	0.684–2.382
N0/N1 + 2	0.525	1.324	0.558–3.145	0.623	1.240	0.526–2.926

DFS, disease-free survival; OS, overall survival; HR, hazard ratio; CI, confidence interval.
